# T-614 attenuates knee osteoarthritis via regulating Wnt/β-catenin signaling pathway

**DOI:** 10.1186/s13018-021-02530-2

**Published:** 2021-06-22

**Authors:** Shan Cong, Yan Meng, Lingrui Wang, Jiao Sun, Ta bu shi·Nu er xia ti, Li Luo

**Affiliations:** 1 Department of Rheumatism and Immunology, First Affiliated Hospital of Xinjiang Medical University, Xinjiang, 830017 P.R. China; 2 Department of Rheumatism and Immunology, Xinjiang Medical University, Xinjiang, 830017 P.R. China; 3 Department of Rheumatism and Immunology, The Second Affiliated Hospital of Xinjiang Medical University, Xinjiang, 830017 P.R. China

**Keywords:** Knee osteoarthritis, Articular cartilage, Iguratimod, Inflammation, Wnt/β-catenin signaling pathway

## Abstract

**Background:**

The aim of this study was to investigate the effect of Iguratimod (T-614) on rat knee osteoarthritis (KOA) and further to explore its underlying mechanism.

**Methods:**

In this study, papain-induced KOA model was constructed. Hematoxylin and eosin (H&E) staining was conducted to observe the pathological changes of cartilage tissue and Mankin scoring principle was used for quantitative scoring. Transmission electron microscopy (TEM) was applied to observe the ultrastructure of cartilage tissue. ELISA was used to measure the levels of matrix metalloproteinase 13 (MMP-13) and inflammatory factors (interleukin (IL)-6 and tumor necrosis factor a (TNF-a)) in serum. RT-qPCR and immunohistochemistry were conducted to detect mRNA expression and protein expression of key genes in Wnt/β-catenin pathway.

**Results:**

H&E, Mankin scoring, and TEM data confirmed that compared with model group, T-614 significantly improved the degeneration of articular cartilage. Besides, we observed that low, middle, and high doses of T-614 could decrease the levels of MMP13, TNF-α, and IL-6 in serum to different degrees. Mechanically, T-614 downregulated the mRNA and protein expression of β-catenin and MMP13 in cartilage tissue via a dose-dependent manner, and on the contrary upregulated the mRNA and protein expression of glucogen synthase kinase-3 beta (GSK-3β).

**Conclusion:**

Our results suggested that T-614 can reduce the level of its downstream target gene MMP-13 and downregulate the expression of inflammatory cytokines TNF-α and IL-6 by regulating the Wnt/β-catenin signaling pathway, thereby inhibiting joint inflammation and controlling KOA degeneration of articular cartilage.

## Background

Knee osteoarthritis (KOA) is an inflammatory disease occurring in human joints and their surrounding tissues. The process is slow, and more in middle-aged and elderly people [1, 2]. Its clinical manifestations are joint dysfunction and joint deformity, causing pain and limited movement, which greatly reduces the quality of life of patients [3]. According to reports, the incidence of KOA symptoms among people over 65 years of age in China is higher (19.4%), among which females are higher than males, 10.3% and 5.7%, respectively [4]. In addition, with the aging and increasing obesity of the world’s population, KOA ranks 11th in global disability and 38th in disable-adjusted life years [5]. Currently, the treatment of KOA is mainly drug interventions, such as hormones and oral COX inhibitors, but its therapeutic efficacy is still limited, and there are many drug-related adverse events [5, 6]. For patients who do not respond to medication, surgery may be used. Although surgical treatment has a good effect in improving patients’ pain, nearly half of patients still have long-term pain after surgery, and some patients need a second surgery [7, 8]. Therefore, it is urgent to seek a new treatment method or new therapeutic drugs for KOA.

Iguratimod (N-[7-[(methanesulfonyl)amino]-4-oxo-6-phenoxy-4H-1-benzopyran-3-yl] formamide, T-614) is a small-molecule targeted drug independently developed in China, which has the functions of anti-inflammatory and anti-rheumatic drugs for immune regulation and inhibiting joint destruction. T-614 was originally reported as a selective inhibitor of cyclooxygenase (COX-2), which inhibits the activity of nuclear factor-κB and various inflammatory cytokines [9]. For example, Hou et al. demonstrated that T-614 reduces severe acute pancreatitis by inhibiting NLRP3 inflammasome and NF-κB pathway [10]. In clinical trials, it was also observed that T-614 significantly improved rheumatoid arthritis symptoms [11]. In addition, Du et al. revealed that T-614 can not only improve the symptoms of rheumatoid arthritis, but also prevent bone destruction [12]. However, the research on the mechanism of T-614 acting on cartilage tissue has been poorly elucidated.

Wnt signaling is a growth control pathway that can regulate many biological processes ranging from development and evolution to adult homeostasis. Wnt signaling includes two branches, canonical (β-catenin-dependent activity) and non-canonical (β-catenin-independent activity) Wnt pathways. β-catenin is composed of a central region, including 12 imperfect Armadillo repeats, which are flanked by different domains in N- and C-terminus, respectively [13]. It acts as a crucial nuclear effector of the canonical Wnt pathway. Dysregulation of Wnt/β-catenin signaling, which is necessary for many vital biological processes, such as embryonic development, organogenesis, tissue regeneration, hematopoiesis, cell survival, cellular proliferation and differentiation, and stem cell renewal [14], is associated with many diseases, including osteoporosis, neurodegenerative diseases, cardiovascular diseases, and numerous human malignancies [15, 16]. However, how Wnt/β-catenin signaling affects KOA and its regulatory mechanisms have not been extensively studied.

Hence, in this study, we used T-614 to intervene in the KOA animal model to observe its effects on inflammation, Wnt/β-catenin signaling and cartilage tissue, and further to explore the effects of T-614 on KOA rats at the tissue and molecular level. This will provide theoretical basis and support for the clinical use of T-614 in the treatment of KOA.

## Materials and methods

### Animals and groups

**Fig. 1:**
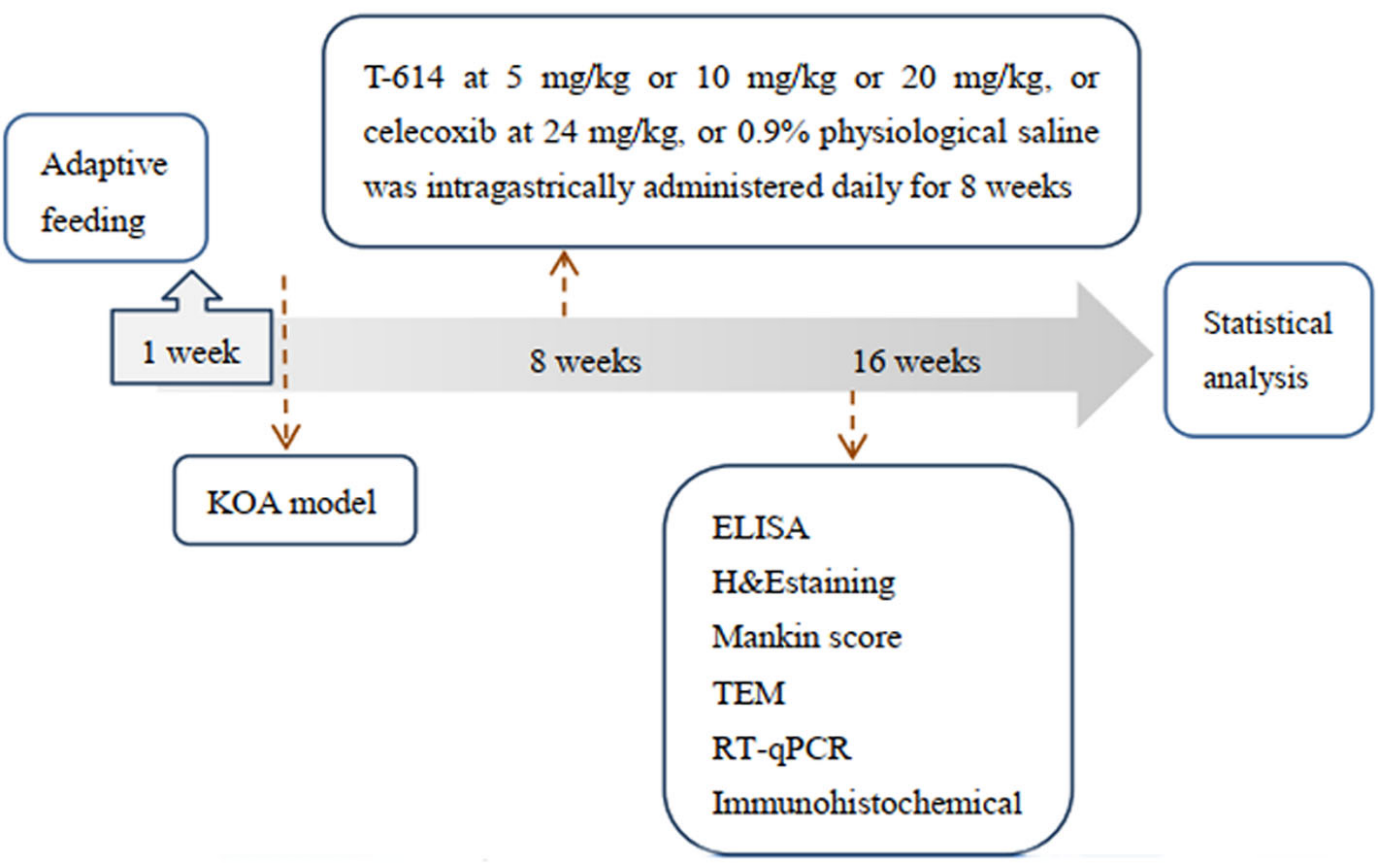
The flow chart of this study

Totally, 60 male Sprague-Dawley (SD) rats weighting 180 to 220 g were purchased from the laboratory of Xinjiang Medical University (qualified number: SCXX (Xin) 2019-0016). All animals were allowed access to water and standard food ad libitum. After adaptive feeding for 1 week, the rats were randomly divided into 6 groups: control group (*n* = 10), model group, *n* = 10), positive control group (*n* = 10), T-614 low-dose group (*n* = 10), T-614 middle-dose group (*n* = 10), and T-614 high-dose group (*n* = 10). The rats in the control group were injected with 0.15 mL of physiological saline (0.9%) in the right knee joint cavity, and the rats in the other groups were all established OA models. After 8 weeks of modeling, the control group and the model group were intragastrically given physiological saline (0.9%). The positive control group was intragastrically administered with 24 mg/kg celecoxib. The T-614 low-dose group was intragastrically administered with T-614 5 mg/kg every daily. T-614 middle-dose group was intragastrically administered with T-614 10 mg/kg every daily. The T-614 high-dose group was intragastrically administered with T-614 20 mg/kg daily. Each group was continuously administered for 8 weeks. This study was approved by the Animal Ethics Committee of Xinjiang Medical University and the approval number is Institutional Animal Care and Use Committee (IACUC)-1902019. All experiments were carried out in accordance with the National Institute of Health Guide for the Care and Use of Laboratory Animals. Figure 1 is the flow chart of this study.

### Establishment of KOA model

SD rats were anesthetized by intraperitoneal injection of 1% ketamine (50 μg/g), and then were fixed on the board in supine position. After cutting the leg hair in the 1 cm area around the knee cavity of the right hind limb, it was disinfected with iodine complex. The knee joint of the rats was flexed 45°, and the needle (28) was pierced in the direction of the intercondylar fossa with the knee eye on the outer edge of the white patella tendon under the patella as the needle entry point. A 1-mL syringe was used to inject 0.15 mL of the mixed solution (2% papain: 0.03 mol/L l-cysteine = 2:1) into the right knee joint cavity, and the injection was repeated once 4 days and 7 days later.

### Measurement of MMP-13, interleukin (IL)-6, and TNF-a levels by ELISA

First, blood was collected from the abdominal aorta and placed at room temperature for 20 min. After the blood coagulated naturally, the supernatant was collected by high-speed centrifugation. The levels of IL-6, MMP13, and TNF-α in peripheral blood of rats were detected by ELISA kit (USCN Business Co., Ltd. Wuhan). Each experiment was repeated three times according to the literature standard. The details are as follows: ELISA kit for IL-6 (Product No.: SEA079Ga; detection range 15.6–1000 pg/mL; sensitivity: the minimum detectable dose of this kit is typically less than 5.5 pg/mL; intra-assay CV < 10%; inter-assay CV < 12%). ELISA kit for MMP13 (Product No.: SEA099Hu; detection range 0.312–20 ng/Ml; sensitivity: the minimum detectable dose of this kit is typically less than 0.113 ng/mL; intra-assay CV < 10%; inter-assay CV < 12%). ELISA kit for TNF-α (Product No.: SEA133Si; detection range 7.8–500 pg/mL; sensitivity: the minimum detectable dose of this kit is typically less than 2.7 pg/mL; intra-assay CV < 10%; inter-assay CV < 12%).

### RT-qPCR

The articular cartilage of 8 rats from each group was placed in a clean mortar, and liquid nitrogen was then added to grind into powder. TRIpure reagent (Invitrogen, USA) was used to isolate the total RNA and PrimeScript RT kit (TaKaRa, Otsu, Japan) was used for reverse transcription. The cDNA was used as the template and the PCR reaction was performed according to the kit instructions. The PCR reaction conditions were as follows: 95 °C for 15 min; 40 cycles of 95 °C for 20 s, 56 °C for 20 s, and extension at 72 °C for 1 min. Each experiment was repeated three times. β-actin was controlled as housekeeping gene. The primers are shown in Table 1.

**Table 1:** Primer sequences of PCR

Gene Forward (5′-3′) Reverse (3′-5′)		
β-catenin	CTTCCAGACACGCCATCATG	CACACAGAGTACTTGCGCTC
MMP13	CCTAAGCACCCCAAA ACACC	TGGTGATGGCGTAGAACAGT
GSK-3β	ATGTATGGTCTGCAGGCTGT	GGATGTGCCTTGATTTGGGG
β-actin	TCTTCCAGCCTTCCTTCCTG	CACACAGAGTACTTGCGCTC

*MMP13* matrix metalloproteinase 13, *GSK-3β* glucogen synthase kinase-3 beta

### Hematoxylin and eosin (H&E) staining

After the rat modeling and administration, the tibial plateau and femoral condyle cartilage were taken for pathological observation. Briefly, the samples from 5 rats were fixed in 4% paraformaldehyde for 24 h. Subsequently, the samples were embedded in paraffin after decalcification and cut into 5 μm sections. After routine dewaxing and dehydration, sections were soaked with hematoxylin for 5 min and then with eosin for 5 min.

### Mankin score

After H&E staining, sections were scored using the Mankin scoring principle by two experienced blind observers [17], which ranged from 0 to 14 (total score), representing the total score for the severity of cartilage structure, chondrocytes, and tidal line damage. Among them, 1 to 5 was classified as early OA, 6 to 9 as middle OA, and 10 to 14 as late OA (Table 2).

**Table 2:** Mankin scoring criteria

Structure	Standard	Score
Structure	Normal	0
	Surface irregularities	1
	Pannus and surface irregularities	2
	Clefts to transitional zone	3
	Clefts to radial zone	4
	Clefts to calcified zone	5
	Complete disorganizations	6
Cells	Normal	0
	Diffuse hypercellularity	1
	Cloning	2
	Hypocellularity	3
Safranin-O staining	Normal	0
	Slight reduction	1
	Moderate reduction	2
	Severe reduction	3
	No dye noted	4
Tidemark integrity	Intact	0
	Crossed by blood vessels	1

### Transmission electron microscopy (TEM)

After modeling and administration, 5 rats in each group were anesthetized and the articular cartilage was collected. Then the cartilage was rinsed repeatedly with normal saline, and the surface of the cartilage was observed to be free of dirt, which could be placed under TEM. After a certain amount of air was extracted from the closed dark room where the specimen was to be placed, the cartilage surface was observed and figures were taken.

### Immunohistochemical analysis

Endogenous peroxidase activity within the sections was quenched by incubating the sections with 3% H_2_O_2_ for 15 min after dewaxing and hydration. Then, the cartilage tissues were incubated with primary antibodies directed against β-catenin (ab16051, Abcam), MMP13 (ab39012, Abcam), and GSK-3β (ab93926, Abcam). On the following day, the tissues were washed with PBS and incubated with secondary antibody anti-Rabbit lgG (MaiXin Bio, China). In the negative controls, the primary antibody was replaced by PBS. They were counterstained with DAB (KT1009a, Abgent) and sealed with glass slide by resin. Image Pro Plus 6.0 was used to measure the immunohistochemical staining positive area and cumulative optical density (IOD), and IOD (SUM)/Area (SUM) was used as the final measurement value.

### Statistical analysis

SPSS 17.0 (IBM, New York, USA) was applied to analyze all data and the data presented as mean ± standard deviation. The data normality was tested by the Shapiro-Wilk test. Differences among multiple groups were statistically analyzed using one-way ANOVA and post hoc comparisons (Dunnett’s test). Values of *P* < 0.05 were considered statistically significant.

## Results

### The histopathological changes of articular cartilage and Mankin score

**Fig. 2:**
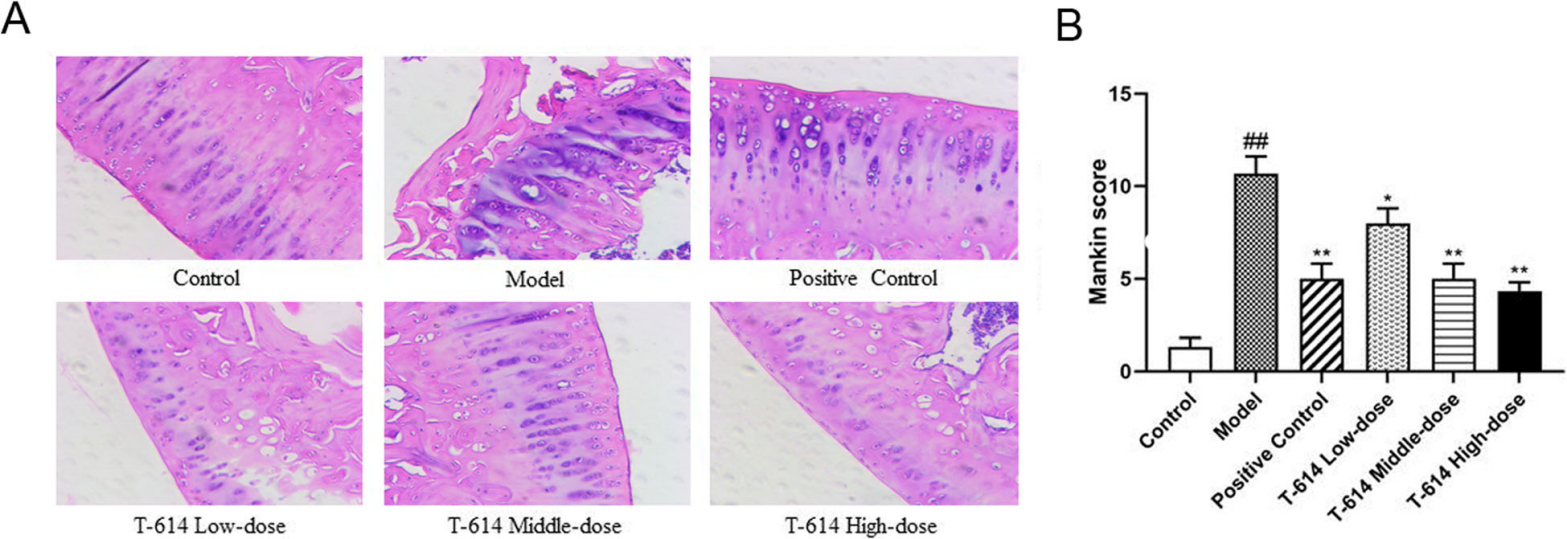
The histopathological changes of articular cartilage and Mankin score. **A** Following treatment with or without T-614, histopathological alterations were evaluated by H&E staining. **B** Following H&E staining, the histological grading was evaluated by a modified Mankin scoring principles. ^##^ indicated compared with control group, (*P* < 0.01); ^*^ and ^**^ indicated compared with model group, (*P* < 0.05 and *P* < 0.01). H&E staining, hematoxylin and eosin staining

As presented in Fig. 2A, the whole sections of rats in the control group were uniformly stained, with clear cartilage structure, smooth surface, orderly cell arrangement, and complete tidal line. In the model group, chondrocytes were disordered, cytoplasm was swollen, the number of chondrocytes was significantly reduced, and the tide line was completely detached. In the T-614 low-dose group, the cartilage structure was defective, the cell arrangement was disordered, and the tide line was broken, while in T-614 middle-dose group, the cartilage structure was fussy, the chondrocytes were disordered and aggregated, and the tidal line was slightly incomplete. However, in the T-614 high-dose group, we found that the cartilage structure was significantly improved compared with the model group and the T-614 low–middle-dose group, which showed some irregular cartilage fissures, occasionally swelling of chondrocytes and complete tide lines.

To more accurately observe the changes of cartilage tissue in each group, Mankin score was used for quantitative scoring. As presented in Fig. 2B, the Mankin score in the model group was 10.87 ± 0.76, which was significantly increased compared to the control group (1.23 ± 0.52, *P* < 0.01), suggesting that the early KOA model was successfully established. After treatment with T-614, the Mankin scores of the different drug groups were significantly reduced compared with the model group, presenting a dose-dependent pattern. In addition, the Mankin score of the T-614 middle-dose group (4.92 ± 0.56) and T-614 high-dose group (4.75 ± 0.37) was similar to that of the positive control group (4.98 ± 0.65), indicating that T-614 significantly improved KOA.

### The microstructural changes of articular cartilage

**Fig. 3:**
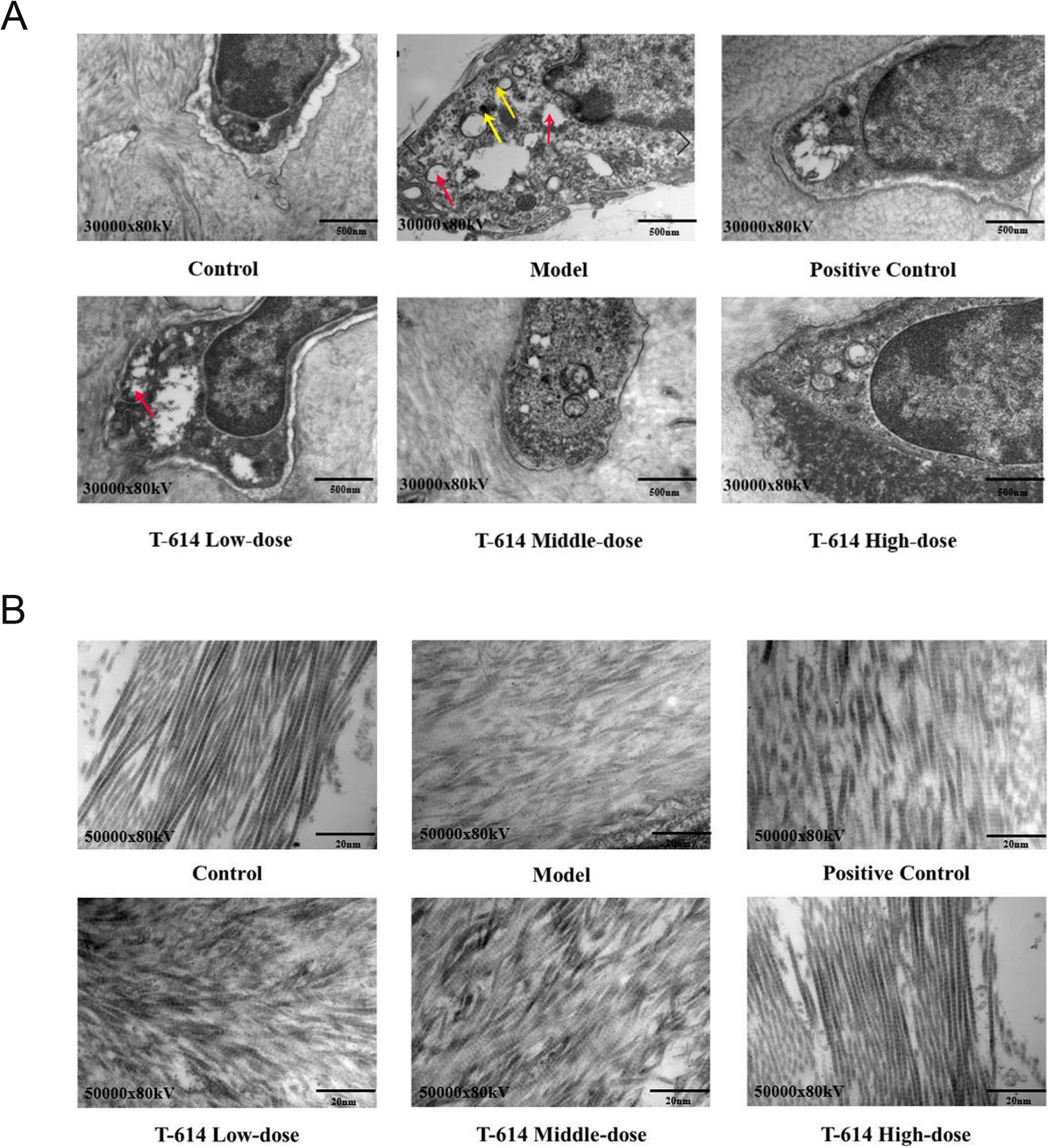
The microstructural changes of articular cartilage. The morphological changes in the cartilage were observed under a microscope and images were acquired at a magnification of × 30,000 (**A**) and × 50,000 (**B**); the yellow arrows represent exfoliated particles and red arrows represent vacuole-like structure

Under TEM, the chondrocytes in the control group had normal morphology and intact cell membranes. Many microvilli of different lengths were seen on the surface, no obvious shedding particles were seen, and the collagen fiber layer was densely arranged. Compared with the control group, chondrocyte capsule granules were prolapse, contents of chondrocyte were swollen and enlarged, and the membrane structure appeared marginal voids or vacuole-like structures in the model group. Besides, the collagen fibrous layer was loose and broken. In the T-614 low-dose group, particle shedding and vacuole-like structure were improved, while the collagen fiber layer remained disordered. In the T-614 middle-dose group, there were few vacuoles and restored microvilli, but the collagen fibrous layer was disordered. Notably, the microvilli recovered significantly in the T-614 high-dose group, with a significant decrease in exfoliated particles and vacuole-like structures, and compact arrangement of collagen fiber layers (Fig. 3A, B).

### The expression of MMP13 and cytokines TNF-α and IL-6 in serum

**Fig. 4:**
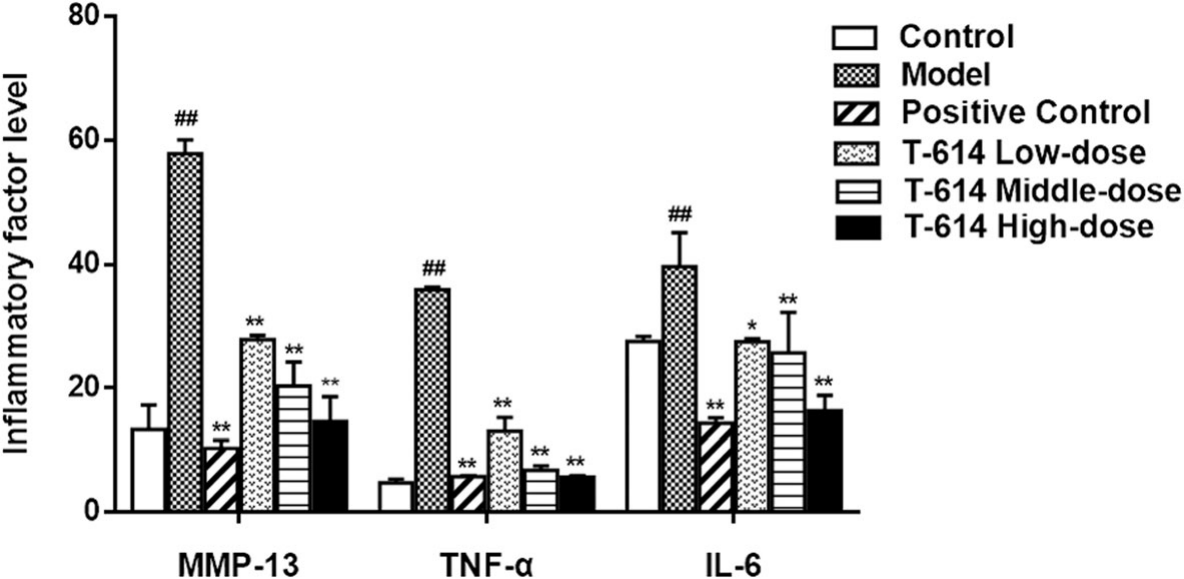
The expression of MMP13 and cytokines TNF-α and IL-6 in serum. Following treatment with or without T-614, the expression of MMP13 and cytokines TNF-α and IL-6 in serum were detected by ELISA. ^##^ indicated compared with control group (*P* < 0.01); ^*^ and ^**^ indicated compared with model group (*P* < 0.05 and *P* < 0.01)

As shown in Fig. 4, the levels of MMP13, TNF-α, and IL6 in serum of the model group were significantly higher than those in the control group (4.5-fold increase, 7-fold increase, and 1.5-fold increase, respectively). Additionally, compared with the model group, the levels of MMP13, TNF-α, and IL6 were significantly reduced in the positive control group and in different concentrations of T-614 groups. Among them, the T-614 high-dose group (decrease 76%, 85%, and 59%) and positive control group (decrease 82%, 85%, and 64%) reduced the most obvious (*P* < 0.01).

### The mRNA expression of β-catenin, MMP13, and GSK-3β in articular cartilage

**Fig. 5:**
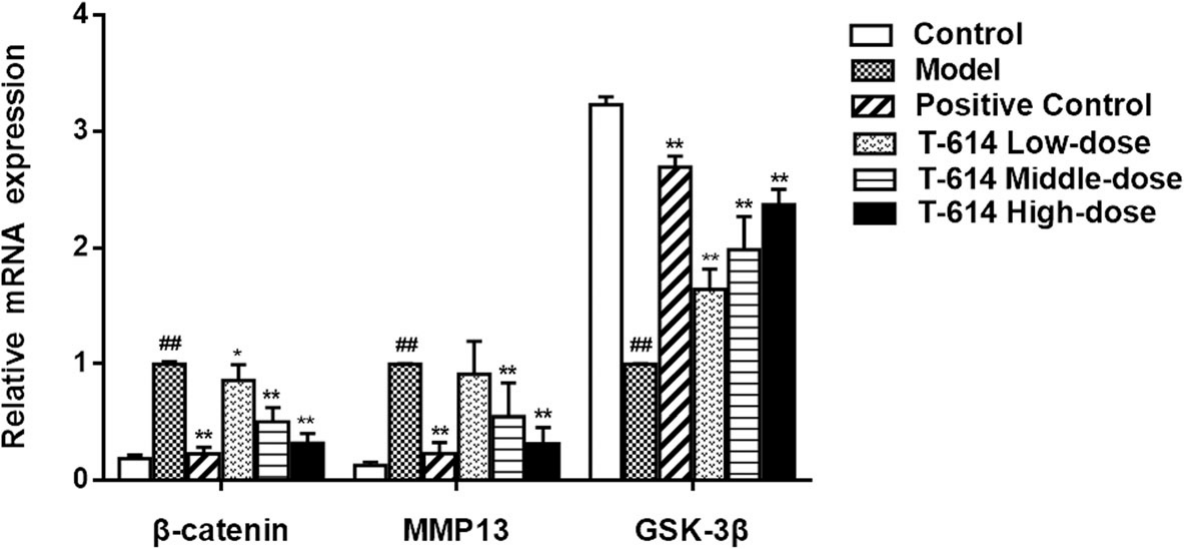
The mRNA expression of β-catenin, MMP13, and GSK-3β in articular cartilage. Following treatment with or without T-614, the mRNA expression of β-catenin, MMP13, and GSK-3β in articular cartilage. ^##^ indicated compared with control group (*P* < 0.01); ^*^and ^**^indicated compared with model group (*P* < 0.05 and *P* < 0.01)

Compared with the control group, the levels of β-catenin and MMP13 mRNA in articular cartilage of rats in model group were evidently increased (5-fold increase and 7-fold increase, respectively). On the contrary, GSK-3β mRNA expression was significantly decreased (67%, *P* < 0.01). After treatment with T-614, the above gene expression pattern changed. Specifically, compared with the model group, whether in low, middle, and high dose of T-614 groups, the level of β-catenin was significantly decreased, while the expression of GSK-3β was significantly increased (*P* < 0.05 or *P* < 0.01). For MMP13, except for the T-614 low-dose group, there was no significant difference, and the other two groups were both significantly reduced (*P* < 0.01, Fig. 5). In addition, we observed that the changes of the above three genes were consistent between the T-614 high-dose group and the positive control group.

### The protein expression of β-catenin, MMP13, and GSK-3β in articular cartilage

**Fig. 6:**
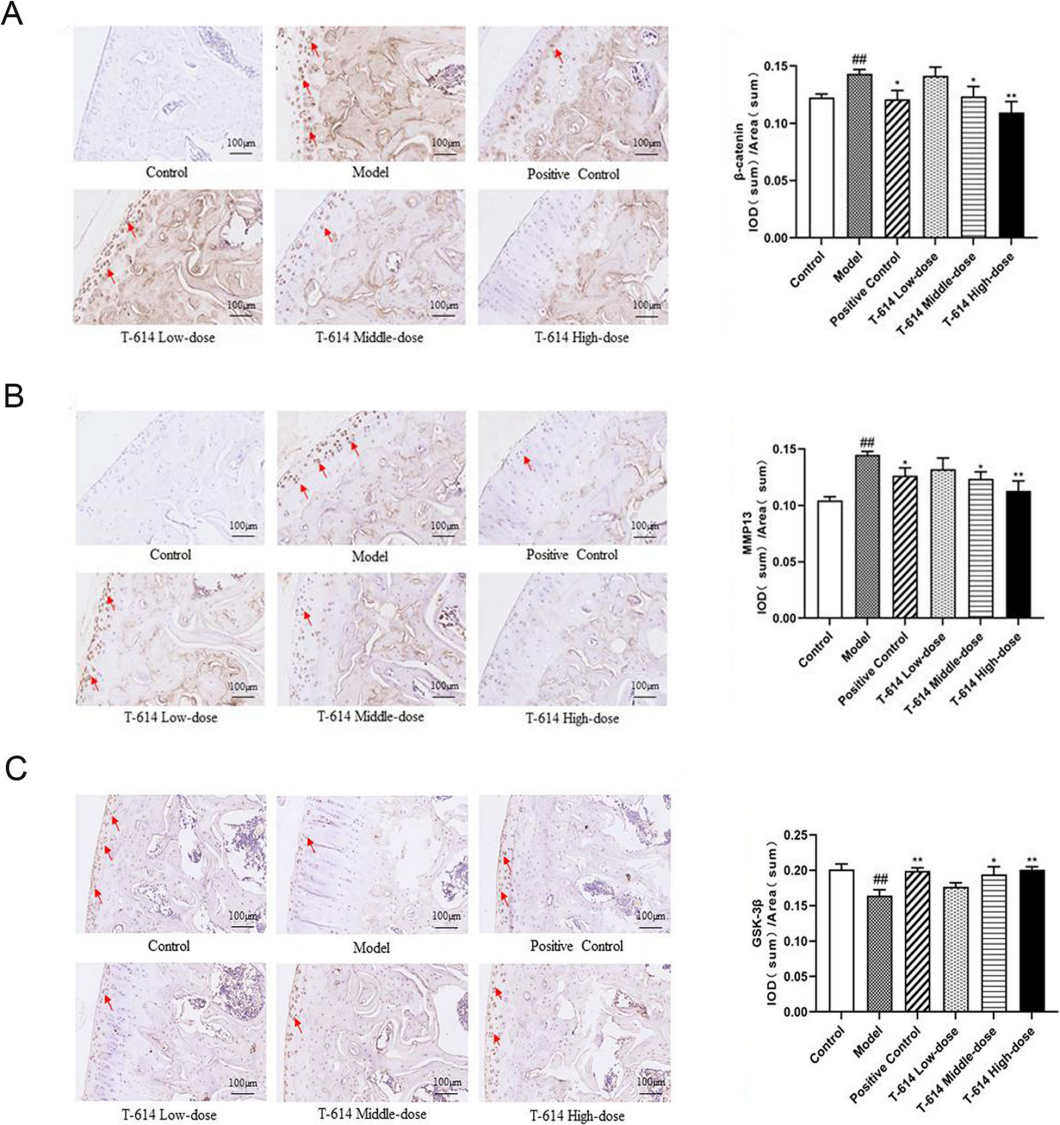
The protein expression of β-catenin, MMP13, and GSK-3β in articular cartilage. Following treatment with or without T-614, the protein expression of β-catenin (**A**), MMP13 (**B**), and GSK-3β (**C**) in articular cartilage. ^##^ indicated compared with control group (*P* < 0.01); ^*^and ^**^ indicated compared with model group (*P* < 0.05 and *P* < 0.01). The red arrows represent the positive cell nucleus with deep yellow staining

Immunohistochemical positive reactions in the cartilage presented different shades of yellow-brown staining. As the color deepens, the positive reaction gradually increases. In Fig. 6A, B, the positive reaction of MMP13 and β-catenin in the cartilage of rats in the control group was not obvious, and the chondrocyte nucleus was dark blue. Conversely, GSK-3β presented a strong positive expression, showing a large area of dark brown staining reaction (Fig. 6C). In the model group, MMP13 and β-catenin were strongly positive, mostly located around chondrocytes, while GSK-3β was not obvious. Interestingly, after treatment with different concentrations of T-614, the protein levels of MMP13 and β-catenin were gradually decreased, and GSK-3β was gradually increased in a dose-dependent manner.

The quantification results of image Pro Plus showed that compared with the control group, the protein expression of β-catenin and MMP13 in model group were remarkably increased (12% and 35%, *P* < 0.01), while the expression of GSK-3β was significantly decreased (22%, *P* < 0.01). Compared with the model group, the β-catenin and MMP13 protein expression in the positive control group and T-614 group (middle-dose and high-dose) were significantly decreased, while GSK-3β protein levels were significantly increased (25%, 20%, and 25%), which was consistent with the mRNA expression pattern (Fig. 6A–C).

## Discussion

According to reports, the incidence of KOA symptoms among people over 65 years of age in China is higher (19.4%), among which females are higher than males, 10.3% and 5.7%, respectively. Narrower femurs, thinner patellae, and greater angles of quadriceps and differences in the size of tibial condyles make women’s knee anatomy different from men’s, leading to different kinematics, which influences female sex to be more likely to develop OA, ultimately leading to a higher prevalence of OA in women [18]. In view of the different incidences of KOA in different genders, gender has become a problem that needs attention when modeling KOA in rats. Notably, recent data from Wang et al. confirmed no significant differences between the two groups of male and female rats in the use of papain model KOA [19]. In addition, male rats had better physical health indicators than female mice. Moreover, male rats do not have the periodic physiological fluctuations of female rats, their hormone levels are stable, and enzyme activities are relatively stable. Therefore, our study selected male rats for modeling and used this model to explore the effects of T-614 on rat KOA.

The typical pathological change of KOA is the degeneration of articular cartilage, and the function of cartilage tissue is mainly provided by the ordered network structure in the extracellular matrix (ECM) [20]. As a carrier for chondrocytes to absorb nutrients and transmit signals, the metabolic balance of ECM maintains the biological properties of articular cartilage. When ECM is excessively degraded, chondrocytes lose the environment they depend on for survival, which will accelerate the apoptosis of chondrocytes, leading to aggravation of KOA articular cartilage lesions [21]. In the current study, H&E staining showed disordered arrangement of articular chondrocytes, swelling of cytoplasm, significantly reduced number of chondrocytes, and complete removal of tide line in model group. Under TEM, chondrocyte capsule granules were prolapsed, the contents were swollen and enlarged, the membrane structure had marginal voids or vacuole-like structures, and the collagen fiber layer was loose and broken. Notably, after treatment with different doses of T-614, the cartilage tissue structure improved more significantly as the dose of the drug increased. Under TEM, the chondrocyte microvilli were obviously recovered, the shedding particles and vacuole-like structure were significantly reduced, and the collagen fiber layer was tightly arranged. The above pathological changes were consistent with previous studies [18, 22]. Taken together, our data suggested that T-614 may alleviate KOA progression by improving articular cartilage structure.

The most conspicuous biochemical change in KOA cartilage is the loss of proteoglycan and collagen type II. Matrix metalloprotease (MMP)-13 is considered as a well-characterized key player in cartilage biology and KOA pathology due to its capacity to degrade collagens type II and a wide range of other matrix components [23]. The gene and protein expression of MMP-13 increases with severity of cartilage degeneration [24]. These observations support the concept that MMP-13 reflects an intrinsic process of cartilage degradation in KOA. Interestingly, we found a significant increase in serum MMP13 in the model group, which was consistent with previous studies. In an inflammatory environment, the levels of pro-inflammatory cytokines such as TNF-α and IL-6 increased significantly. Among them, IL-6 can inhibit the synthesis of proteoglycans and type II collagen, while TNF-α promoted their catabolism, thereby promoting the expression of matrix metalloproteinases including MMP-13, which ultimately lead to ECM decomposition [21, 25, 26]. Attur et al. found a subgroup of KOA patients in which a gene overexpression of inflammatory cytokines is present (IL-1β and IL-8) [27]. Correspondingly, the serum levels of IL-6 and TNF-αin model group were significantly increased in this study. In addition, in exploring drug relief for KOA, Yu et al. demonstrated that Sivelestat sodium hydrate improved post-traumatic knee osteoarthritis in a rat model with reduced inflammatory cytokines [28]. Interestingly, our research also reduces inflammation response with T-614. Collectively, the above data revealed that T-614 can reduce the level of serum IL-6 and TNF-α, inhibit the secretion of serum MMP-13, and control the degradation of articular cartilage matrix, thereby alleviating the progression of KOA.

Several lines of recent evidence suggest that Wnt/β-catenin signaling may play an important role in regulating the pathogenesis of KOA and other forms of arthritis. Liu et al. showed that exosomes extracted from platelet-rich plasma alleviate KOA by promoting chondrocyte proliferation and inhibiting chondrocyte apoptosis through the Wnt/β-catenin signaling pathway [29]. He et al. confirmed that costunolide inhibits matrix metalloproteinases expression and osteoarthritis via the Wnt/β-catenin signaling pathway [30]. The canonical Wnt pathway triggers its signaling within cells through regulating intracellular β-catenin levels and subcellular localization of β-catenin. In the Wnt signaling pathway, when β-catenin accumulates to a certain concentration in the cell, the excess β-catenin enters the nucleus, binds to T cytokines/lymphatic enhancers, and then activates Wnt/β-catenin transcription factors to promote the expression of downstream target genes such as MMP-13 [31]. In the absence of Wnt proteins, the β-catenin levels are kept in a steady state. Excess β-catenin is phosphorylated by glycogen synthase kinase-3β (GSK-3β) and degraded by ubiquitination and proteasome pathways [32]. Therefore, effective regulation of the Wnt/β-catenin signaling pathway is a key pathway to inhibit the expression of MMP-13, and β-catenin and GSK-3β play a crucial role in maintaining the balance of this signaling pathway. In this study, the levels of MMP-13, β-catenin gene, and protein in the cartilage of the model group were significantly increased; on the contrary, the expression of GSK-3β was significantly decreased, suggesting that high concentration of β-catenin can activate the Wnt/β-catenin pathway and promote the expression of MMP13, which leads to the degradation of cartilage matrix, and finally causes the degeneration of articular cartilage. The results are consistent with those of Shu et al. [33]. Notably, after the addition of T-614, it reversed the expression of these factors in a dose-on-dose manner. Taken together, we confirmed that T-614 may regulate the Wnt/β-catenin signaling pathway, promote the phosphorylation of GSK-3β, reduce the level of β-catenin in the nucleus, thereby inhibit the production of MMP-13, and ultimately affect the pathology of the articular cartilage matrix.

This study has some limitations. This study is a single nodal drug target, and further analysis of the efficacy of T-614 in the treatment of KOA at different time nodes is needed. In addition, the regulatory mechanisms in vivo are complex, and we still need to further explore whether other possible mechanisms, such as OPG/RANKL/RANK, are involved in the regulation of T-614 treatment on KOA. This study observes the pathological changes in rats 8 weeks after modeling, which is equivalent to the late stage of KOA, and we can provide corresponding treatment plans for this stage according to the pathological changes at this stage.

## Conclusion

In this study, we concluded that T-614 can maintain the cartilage matrix homeostasis environment, protect the articular cartilage, and have the effect of treating KOA. The protective mechanism may be achieved by regulating key regulatory factors expression in the Wnt/β-catenin signaling pathway such as β-catenin and GSK-3β, inhibiting the production of inflammatory cytokines such as IL-6 and TNF-α, and reducing the level of MMP13 in articular cartilage.

## Data Availability

All data generated or analyzed during this study are included in this published article.

## Abbreviations

KOA: Knee osteoarthritis
HE: Hematoxylin and eosin
TEM: Transmission electron microscopy
MMP-13: Matrix metalloproteinase 13
IL-6: Interleukin
GSK-3β: Glucogen synthase kinase-3 beta
